# Comparative genomics of *Cryptococcus neoformans* var. *grubii* associated with meningitis in HIV infected and uninfected patients in Vietnam

**DOI:** 10.1371/journal.pntd.0005628

**Published:** 2017-06-14

**Authors:** Jeremy N. Day, Seet Qihui, Lam Tuan Thanh, Phan Hai Trieu, Anh Duong Van, Nha Hoang Thu, Tran Thi Hong Chau, Nguyen P. H. Lan, Nguyen Van Vinh Chau, Philip M. Ashton, Guy E. Thwaites, Maciej F. Boni, Marcel Wolbers, Niranjan Nagarajan, Patrick B. O. Tan, Stephen Baker

**Affiliations:** 1 Oxford University Clinical Research Unit, Wellcome Trust Major Overseas Programme Viet Nam, Ho Chi Minh City, Vietnam; 2 Centre for Tropical Medicine, Nuffield Department of Medicine, University of Oxford, Oxford, United Kingdom; 3 Genome Institute of Singapore, Singapore; 4 Hospital for Tropical Diseases, Ho Chi Minh City, Viet Nam; 5 The London School of Hygiene and Tropical Medicine, London, United Kingdom; University of California San Diego School of Medicine, UNITED STATES

## Abstract

The vast burden of cryptococcal meningitis occurs in immunosuppressed patients, driven by HIV, and is caused by *Cryptococcus neoformans* **var. *grubii***. We previously reported cryptococcal meningitis in Vietnam arising atypically in HIV uninfected, apparently immunocompetent patients, caused by a single amplified fragment length polymorphism (AFLP) cluster of *C*. *neoformans* **var. *grubii*** (VNIγ). This variant was less common in HIV infected individuals; it remains unclear why this lineage is associated with apparently immunocompetent patients. To study this host tropism we aimed to further our understanding of clinical phenotype and genomic variation within Vietnamese *C*. *neoformans* **var. *grubii***. After performing MLST on *C*. *neoformans* clinical isolates we identified 14 sequence types (STs); ST5 correlated with the VNIγ cluster. We next compared clinical phenotype by lineage and found HIV infected patients with cryptococcal meningitis caused by ST5 organisms were significantly more likely to have lymphadenopathy (11% vs. 4%, *p* = 0.05 Fisher’s exact test) and higher blood lymphocyte count (median 0.76 versus 0.55 X10^9^ cells/L, *p* = 0.001, Kruskal-Wallis test). Furthermore, survivors of ST5 infections had evidence of worse disability outcomes at 70 days (72.7% (40/55) in ST5 infections versus 57.1% (52/91) non-ST5 infections (OR 2.11, 95%CI 1.01 to 4.41), *p* = 0.046). To further investigate the relationship between strain and disease phenotype we performed genome sequencing on eight Vietnamese *C*. *neoformans* **var. *grubii***. Eight genome assemblies exhibited >99% nucleotide sequence identity and we identified 165 kbp of lineage specific to Vietnamese isolates. ST5 genomes harbored several strain specific regions, incorporating 19 annotated coding sequences and eight hypothetical proteins. These regions included a phenolic acid decarboxylase, a DEAD-box ATP-dependent RNA helicase 26, oxoprolinases, a taurine catabolism dioxygenase, a zinc finger protein, membrane transport proteins and various drug transporters. Our work outlines the complexity of genomic pathogenicity in cryptococcal infections and identifies a number of gene candidates that may aid the disaggregation of the pathways associated with the pathogenesis of *Cryptococcus neoformans* **var. *grubii***.

## Author summary

Cryptococcal meningitis is a brain infection caused by a yeast, *Cryptococcus neoformans*, and results in an estimated 600 000 deaths each year. Disease usually only occurs in patients who have some problem with their immune systems—most commonly Human Immunodeficiency Virus (HIV) infection. However, it is increasingly recognized that disease can occur, particularly in southeast and east Asia, in patients with apparently normal immune systems (‘immunocompetent’). We previously showed that almost all infections in immunocompetent patients in Vietnam are due to just one small ‘family’ (or lineage) of *Cryptococcus neoformans* var. *grubii*, which we called VNIγ. This is in contrast to disease in HIV infected patients, which can be caused by a number of different families. This suggests that VNIγ strains have an increased ability to cause disease. Here, we define the pattern of disease caused by VNIγ infections compared with other strains in HIV infected patients, and use whole genome sequencing—comparing the entire genetic codes from different strains—to try and understand which genes give the VNIγ family this special ability to cause disease in immunocompetent patients.

## Introduction

Cryptococcosis is a range of disseminated infections caused by yeasts belonging to the genus *Cryptococcus*. Cryptococcosis generally occurs in individuals with cell-mediated immune defects, particularly those infected with Human Immunodeficiency Virus (HIV). As a result of this tropism for the immune-compromised, cryptococcal diseases are commonly fatal. Meningitis is the commonest and most severe disease manifestation, leading to an estimated 600,000 deaths from approximately a million cases per year, globally [1].

Human cryptococcal infections are caused almost exclusively by two species: *Cryptococcus neoformans* (subdivided into 2 varietal forms, *Cryptococcus neoformans* var. *grubii* and *C*. *neoformans* var. *neoformans*), and *Cryptococcus gattii* [2]. The mechanisms that determine the disease prevalence of the cryptococcal species are unknown but associated with host and geographical factors [3]. *C*. *gattii* can be readily isolated from the environment in the tropics and subtropics, but despite comparatively high rates of HIV infection in these regions, human *C*. *gattii* disease is uncommon. *C*. *neoformans* var. *neoformans* is largely restricted to Western Europe, where it accounts for an estimated 25% of cases of cryptococcal meningitis in HIV infected patients [2, 4]. In contrast to *C*. *gattii* and *C*. *neoformans* var. *neoformans*, *C*. *neoformans* var. *grubii* has a global distribution and a devastating impact on the immune-suppressed population [1, 2]. Driven by the high prevalence of HIV infection, the overwhelming majority of cryptococcal meningitis cases occurs in the tropics and sub-tropics and is caused by *C*. *neoformans*.

We previously reported a case series of HIV uninfected patients from Vietnam where the majority of patients did not have a recognized underlying immune suppressive disease [5]. Despite the apparent immune competence of the patients, we found that the majority of infections were caused by *C*. *neoformans* var. *grubii*. We subsequently demonstrated that most cases of disease in HIV uninfected patients in Vietnam were caused by a single amplified fragment length polymorphism (AFLP) defined cluster of *C*. *neoformans* var. *grubii* that we named VNIγ [6]. VNIγ was found to be responsible for 84% of the cases of *C*. *neoformans* var. *grubii* meningitis in HIV uninfected patients and 93% of cases of meningitis in apparently immunocompetent patients, but only 38% of disease in HIV seropositive patients [6]. Furthermore, in the HIV uninfected group, additional underlying diseases were more common in those with non-VNIγ (VNIδ) infections than in those with VNIγ infections.

It is unclear why some *C*. *neoformans* lineages are associated with apparently immunocompetent patients. We hypothesize that the ability to cause disease in these individuals is dependent either on the exploitation of an unidentified immune deficit, or an increased pathogenic potential of the specific lineages. This study has two main aims. First, to determine whether infection with VNIγ strains results in a different clinical phenotype in HIV infected patients, and second to refine our genomic understanding of the variation between Vietnamese *C*. *neoformans* var. *grubii* lineages. We combine MLST profiling of *C*. *neoformans* var. *grubii* with clinical data to describe the phenotypic differences caused by this genotype in HIV infected patients [7]. We then perform genome sequencing and comparative genomics on eight *C*. *neoformans* var. *grubii* to determine the genetic loci that may facilitate an enhanced pathogenic phenotype.

## Methods

### Ethics statement

All clinical studies were approved by the Hospital for Tropical Diseases Ethical Review Board, and either the Oxford Tropical Ethics Committee, or the ethics committee of the Liverpool School of Tropical Medicine. All patients, or their responsible next of kin, gave written informed consent to enter the study.

### *C. neoformans* organisms used in this study

All strains were clinical isolates from HIV infected and uninfected patients from the Hospital for Tropical Diseases, Ho Chi Minh City, Viet Nam enrolled into either a randomised controlled trial of antifungal therapy or a prospective descriptive study [5, 8]. All patients, or their responsible next of kin, gave written informed consent to enter the study. All studies were approved by the Hospital for Tropical Diseases Ethical Review Board, and either the Oxford Tropical Ethics Committee, or the ethics committee of the Liverpool School of Tropical Medicine. All strains were identified using classical mycological methods, sugar assimilation tests (API 32C, BioMerieux, France) and were confirmed as *C*. *neoformans* var. *grubii* molecular group VNI using URA5 RFLP as previously described [9]. Fifty-one strains from HIV uninfected patients were collected between 1996 and 2009. 37 of these were *C*. *neoformans* var. *grubii*, all molecular group VNI. 14 strains were *C*. *gattii*, 13 molecular group VG1 and 1 VG II. The clinical characteristics of the HIV uninfected patients have been published previously—underlying potentially immunosuppressive disease was present in 11 patients [5]. The results of AFLP typing of these strains have been reported previously [6]. The strains from HIV infected patients were the same as those for which we have previously reported AFLP typing. These 100 strains had been randomly selected from the baseline isolates of 238 HIV infected patients who were enrolled into a randomized controlled trial of antifungal therapy in cryptococcal meningitis by 2009 [8]. All 299 strains isolated from patients enrolled in the trial underwent pyrosequencing of 3 MLST loci to divide them into the VNIγ (ST5) versus non-VNIγ lineage. Control strains (*C*. *neoformans* var. *grubii* URA5 RFLP types VNI and VNII) were kindly provided by Associate Professor Wieland Meyer, Westmead Millennium Institute for Medical Research, Sydney, Australia.

### DNA extraction method

Colonies were revived on Sabouraud’s agar at 30°C for 72 h. Single colonies were spread for confluent growth and incubated at 30C for 24 h. Chromosomal DNA was extracted from approximately 0.5 g (wet weight) of yeast cells according to the method described by Wen et al. [10]. The DNA pellet was resuspended in 100 uL of Tris-EDTA (TE) buffer containing 100 ug of RNase.

### URA5 PCR-RFLP typing, MLST, and TAR1 amplification

RFLP analysis of the URA5 gene was carried out according to the methods of Meyer et al.[9]. The final product was separated by electrophoresis on a 3% agarose gel at 100 V for 3 h. RFLP patterns were assigned visually by comparison with known standards. Multi-locus sequence typing was performed according to the ISHAM consensus MLST scheme for the *C*. *neoformans/C*. *gattii* species complex [7]. The seven loci sequenced were: capsule polysaccharide (CAP59), glycerol 3-phosphate dehydrogenase, (GPD1), laccase (LAC1), phospholipase B1 (PLB1), superoxide dismutase (SOD1), orotidine monophosphate pyrophosphorylase (URA5) genes and the intergenic spacer (IGS1) region. PCR primers and thermocycling conditions can be found on the ISHAM website (http://mlst.mycologylab.org). Sequencing was carried out using a 3130xL Genetic Analyzer (ABI). The seven individual loci sequences from the 136 *C*. *neoformans* var. *grubii* strains were concatenated (4,407bp) and aligned to identify 24 polymorphic sites. Allele (AT) and sequence types (ST) were determined using pairwise alignment through the ISHAM *Cryptococcus neoformans* MLST database (mlst.mycologylab.org). VectorNTI (Thermo Fisher, MA, USA) was used for multiple alignments, Bionumerics v7 (Applied Maths, Belgium) with MLST add-in was used for MLST phylogenetic analysis, Bioedit [11] and FigTree (http://tree.bio.ed.ac.uk/software/figtree/) were used for building phylogenetic trees. Phyloviz with the geoBurst algorithm was used to interrogate sequence type structure [12]. TAR1 internal PCR was carried out using primer pairs 5’-CACGAATTGGGACAGGAAGT-3’ and 5’-GAAGAGAAGGAGGCGGAACT-3’ (for further details see Supporting Information S1 Methods). In order to determine the integrity of the VNIγ genotype genomic DNA, amplification of VNIγ-specific DNA (s_1_scaffold4325) was carried out using primer pairs 5’-ATATCAATCGTCGCCTGCTC-3’ and 5’-TTTCTTGGGTTGAGGGTCAG-3’.

### Pyrosequencing method for inferring sequence type

DNA from the MLST alleles IGS1, GPD1 and URA5 were PCR amplified using biotinylated primer pairs targeting the region containing the SNP distinguishing the ST5 genotype. PCR amplifications were performed in 60 μl reactions containing 1 × Hotstart PCR buffer, 1.5 mM of MgCl2, 200 μM of dNTP, 10 pM of each primer, 1.25 Units of Hotstart DNA polymerase (Qiagen, USA) and 3 μl of template DNA. Reactions were cycled once at 95°C for 15 min, followed by 30 cycles of 94°C for 1 min, 60°C for 1 min and 72°C for 1 min, with a final elongation of 72°C for 10 min. All PCR amplifications were visualized on 2% agarose gels prior to pyrosequencing. A pyromark Q96 ID DNA pyrosequencer (Biotage, Sweden) was used to detect the SNPs of interest in each allele as per the manufacturer’s recommendations. PCR amplicons were combined with 56 μl of binding buffer and 4 μl of streptavidin sepharose beads. The resulting mixture was agitated for 5 min before denaturation in denaturation buffer and washing with the Vacuum Prep Tool (Biotage, Sweden). DNA fragments were transferred into a 96-well plate containing 3.5 pmol of sequencing primer in 40 μl of annealing buffer and the DNA sequencing reaction was performed using the Pyro Gold Kit (Biotage, Sweden). The SNP detection mode in the software PyromarkID v1.0 (Biotage, Sweden) was used to analyze sequence data.

### Determining the impact of sequence type on clinical phenotype

All data were from a published randomized controlled trial of combination antifungal therapy for cryptococcal meningitis, Controlled-Trials.com number, ISRCTN95123928 [8]. All patients from the trial with available infecting isolate genotype information were included. Baseline variables (present at study entry) as described in the trial paper were summarized by genotype and compared between the two groups using the Mann-Whitney U test and Kruskal-Wallis test for continuous data and Fisher’s exact test for categorical data.

Early Fungal Activity (EFA), i.e. the rate of clearance of yeast colony forming unit counts from CSF during the first 14 days of antifungal therapy, was estimated in each arm with a mixed effects model of log10-transformed longitudinal fungal count measurements. Comparisons of EFA between ST5 versus non-ST5 were based on a mixed effects model with the fixed intercept term depending on the genotype, the fixed slope term depending on genotype and treatment assignment, and random intercept and slope terms. A Cox regression model was used to determine the impact of sequence type (ST5 versus non-ST5) on survival until 10 weeks (primary endpoint) and six months following study randomisation after adjusting for treatment group, and after adjusting for treatment group, Glasgow coma score at baseline and log10 transformed CSF fungal burden (log_10_ colony forming units/mL) at baseline. The proportion of patients with disability (defined using the Rankin score and two simple questions as described previously) was compared amongst survivors using logistic regression adjusted for the treatment assignment [8]. Statistical analyses were performed with the use of R software, version 3.1.2 [13].

### Genome sequencing, assembly and bioinformatics analyses

1–3 μg genomic DNA from each clinical isolate was used to prepare Illumina paired-end sequencing libraries with Illumina Nextera, sequenced on a HiSeq2000 to deliver approximately 260X coverage The sequencing reads were then assembled by SOAPdenovo [14] and Opera [15]. Genotype-specific DNA sequences, which were found in all the genome assemblies of only one genotype, were identified by alignment using NUCmer [16]. Blastx analysis of the genotype-specific DNA sequences using the NCBI non-redundant protein sequences (nr) database or H99 protein database (*Cryptococcus neoformans var*. *grubii* H99 Sequencing Project, Broad Institute of Harvard and MIT (http://www.broadinstitute.org/) was carried out with a cut-off e-value of 1e-10 [17]. Protein homologs in the H99 genome were identified as proteins with BLASTx hits that had e-value > 1e-10. BlastN analysis of the genotype-specific DNA scaffolds identified the location of these DNA sequences in the H99 genome assembly (Broad Institute). Repeat elements were identified by RepeatMasker (Smit, AFA, Hubley, R & Green, P. *RepeatMasker Open-3*.*0*.) using fungi retrotransposon database (Repbase 17.01) [18]. SNPs were identified using a combination of Stampy and SOAP analysis using *C*. *neoformans var grubii* H99 reference genome (22 SNPs identified by this strategy were verified to be 100% accurate by Sanger sequencing). Indels were identified by Stampy and quality filtered. The location of the SNPs and indels in the genome were predicted using snpEff [19, 20]. For phylogenetic analysis data were mapped to the *C*. *neoformans* H99 reference genome (NCBI accession: NC_026745) using BWA mem v 0.7.13 [21], more than 95% of the genome had high quality mapping for each strain. SNPs were called with GATK v3.3 [22] and filtered with the following thresholds: minimum depth ≥ 5x, ≥ 90% consensus, GQ ≥ 30. Genome positions that had a SNP passing quality thresholds in at least one strain were then extracted using snp-sites and RAxML v8.2.8 (GTR GAMMA model and Lewis ascertainment correction) used to derive a maximum likelihood tree [23]. Data are available at the European Nucleotide Archive (http://www.ebi.ac.uk/ena) under the study code PRJEB17690.

## Results

### Multi-locus sequence typing of *C. neoformans*

Aiming to better define the apparent Vietnamese *C*. *neoformans* var. *grubii* VNIγ tropism for non-immune compromised individuals we performed MLST on seven loci (CAP59, LAC1, PLB1, GPD1, SOD1, URA5 and IGS1) on all available (n = 38) *C*. *neoformans* var. *grubii* isolated from the cerebrospinal fluid (CSF) of HIV uninfected patients with cryptococcal meningitis at our hospital in Ho Chi Minh City (HCMC) in Vietnam [7]. We additionally performed MLST on 96 randomly selected *C*. *neoformans* var. *grubii* strains isolated from HIV infected patients attending the same hospital over the same time period. Two additional reference strains (WM148 (VN1) and WM626 (VN2)) were included; all 136 isolates are described in S1 Table.

The seven loci (4,407 bp in total) from the *C*. *neoformans* var. *grubii* contained 24 polymorphic sites (five singletons). We next determined the allele type (AT) and sequence type (ST), identifying 19 independent ATs, which resulted in 14 STs (S1 Table). ST4 (n = 32), ST5 (n = 65) and ST6 (n = 12) were the most commonly identified. We found two previously unreported ATs at the CAP59 and PLB1 loci in strains BMD1367 and BK55, respectively. Of the 14 STs, five were novel (ST306, ST337, ST338, ST339, and ST340). Each novel ST was comprised of a single strain, four originating from HIV infected patients and one from an HIV uninfected patient.

**Fig 1:**
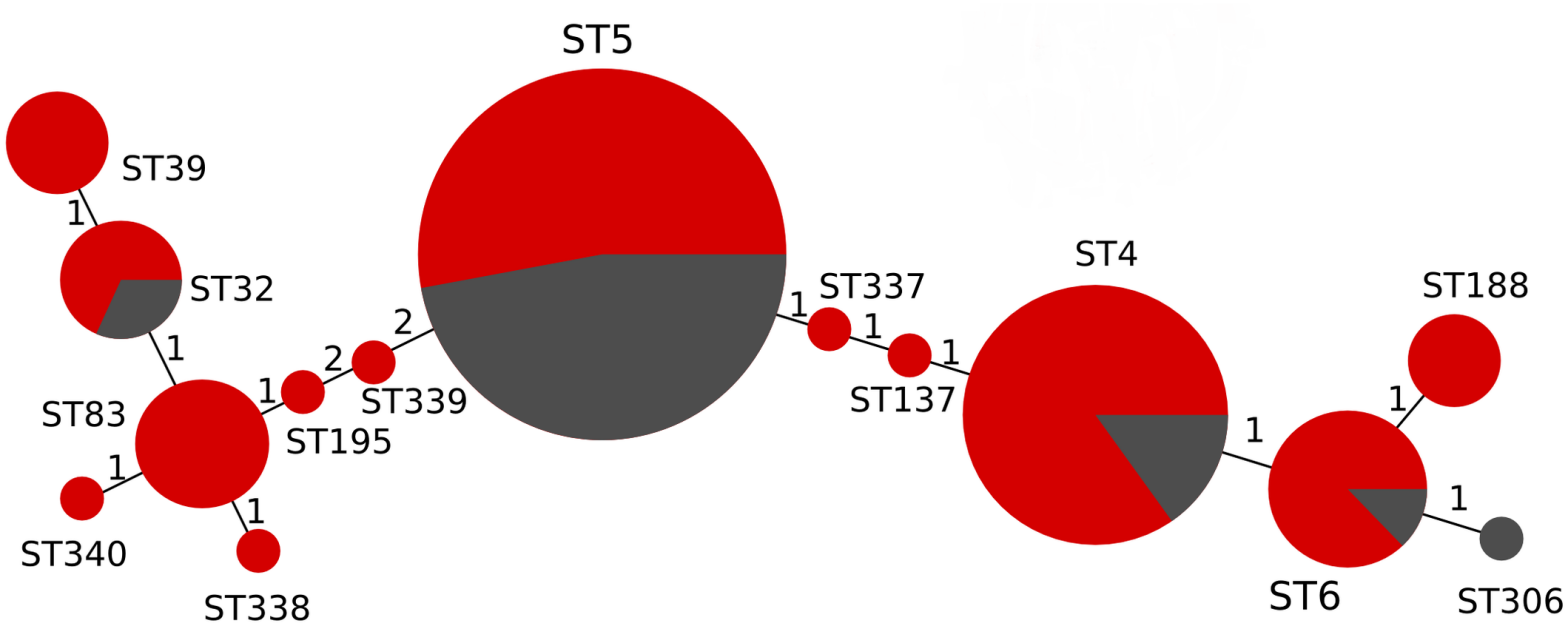
Population structure of Vietnamese clinical isolates (MLST). Minimum-spanning tree of the 14 detected STs and their relative distribution between HIV infected and HIV uninfected patients of 136 Vietnamese clinical isolates of *C*. *neoformans* var. *grubii*. Circle sizes are proportional to the number of isolates; red = isolate from HIV infected patient, grey—isolate from HIV uninfected patient. ST—multi locus sequence type.

Fig 1 shows a minimum spanning tree of the 14 detected STs and their relative distribution between HIV infected and HIV uninfected patients. We found that all ST5 strains precisely correlated with our previously identified AFLP cluster, VNIγ. However, one previously unreported ST (ST337), and one of the eight ST93 strains, was formerly in the VNIγ AFLP cluster. After stratification by HIV status, we found that organisms belonging to ST5 (previously VN1γ) were responsible for 82% (31/38) of the cryptococcal meningitis cases in HIV uninfected patients and 35% (34/98) of the cryptococcal meningitis cases in HIV infected patients (OR 8.2, 95% CI 3.1 to 24.5, *p*<0.001, Fisher’s exact test).

### The clinical phenotype of *C. neoformans* ST5 in HIV infected patients

*C*. *neoformans* ST5 were significantly associated with the immune status of the patient suggesting they may have a different pathogenic potential. Therefore, we aimed to determine whether this ability to infect apparently immune competent patients was associated with any additional ability to affect disease presentation or outcome. As clinical disease phenotype in patients with cryptococcal meningitis is confounded by HIV status we compared the clinical phenotypes of ST5 *C*. *neoformans* var. *grubii* infections against non-ST5 *C*. *neoformans* var. *grubii* in HIV infected individuals only. Using four Single Nucleotide Polymorphisms (SNPs) and two insertion/deletion (indel) sequences within the IGS1, URA5 and SOD1 loci we further screened 290 *C*. *neoformans* var. *grubii* from HIV infected patients enrolled into an anti-fungal RCT in Vietnam [8]. One hundred and three (35.6%) of the *C*. *neoformans* var. *grubii* from this cohort were inferred to be ST5, or within the ST5 complex. The remaining 187 (64.4%) isolates were categorized as non-ST5 strains. The clinical characteristics of patients stratified by ST5 and non-ST5 infecting strains are shown in Table 1.

**Table 1:** Effect of infection with ST5 or non-ST5 *Cryptococcus neoformans* var. *grubii* on clinical phenotype of cryptococcal meningitis in HIV infected patients.

Characteristic	ST5 (N = 103)		Non-ST5 (N = 187		Comparison
	n	Summary statistic	n	Summary statistic	(*p*-value)
**History**					
Duration of symptoms (days)	97	14 (7, 21)	166	13 (7, 20)	0.55
Fever	101	78 (77.2%)	184	138 (75%)	0.78
Headache	102	101 (99.0%)	185	183 (98.9%)	1.00
Neck stiffness	94	64 (68.1%)	176	126 (71.6%)	0.58
Confusion	102	30 (29.4%)	186	54 (29.0%)	1.00
Coma	102	16 (15.7%)	185	16 (8.7%)	0.08
**Findings on examination**					
Weight Kg	101	47.0 (43.0,50.0)	183	47.0 (42.0,50.0)	0.78
Temperature °C	101	37.5 (37.0,38.0)	185	37.5 (37.0,38.0)	0.31
Heart Rate bpm	101	92 (80,100)	185	90 (80,100)	0.57
Glasgow coma score	101		185		0.12
15		11 (10.9%)		10 (5.41%)	
11–14		16 (5.8%)		43 (23.2%)	
≤10		11 (10.9%)		10 (5.4%)	
Cranial nerve lesions	99	21 (21.2%)	186	44 (23.7%)	0.66
Papilledema	94	19 (20.2%)	165	33 (20.0%)	1.00
Visual impairment	90	22 (24.4%)	162	39 (24.1%)	1.00
Neck stiffness	99	70 (70.7%)	185	138 (74.6%)	0.49
Hemiplegia	101	3 (3.0%)	185	9 (4.9%)	0.55
Urinary retention	101	3 (3.0%)	184	6 (3.3%)	1.00
Skin lesions	99	39 (39.4%)	180	59 (32.8%)	0.30
Lymphadenopathy	100	11 (11.0%)	184	8 (4.4%)	**0.045**
Hepato/splenomegaly	101	7 (6.9%)	185	9 (4.9%)	0.59
**Baseline Investigations**					
**Blood**					
CD4 count cells/uL	74	22.0 (9.0,38.5)	142	14.5 (8.0,27.0)	0.053
Blood culture positive	63	39 (61.9%)	104	83 (79.8%)	**0.019**
Haemoglobin	93	12.2 (10.6,13.4)	177	11.8 (10.5,13.2)	0.29
White Cell Count	100	6.3 (4.7,8.8)	181	6.0 (4.3,8.1)	0.19
Lymphocyte count	99	0.8 (0.5,1.0)	181	0.6 (0.4, 0.8)	**0.001**
Blood glucose	93	5.3 (4.9,6.2)	176	5.8 (4.8,6.7)	0.06
**Cerebrospinal fluid**					
Baseline fungal count Log10CFU/ml	78	5.4 (4.7,6.1)	153	5.9 (5.3,6.5)	**0.010**
Raised CSF pressure cm/CSF	83	57 (68.7%)	155	111 (71.6%)	0.66
CSF white cell count cells/uL	91	35.0 (12.0, 97.5)	169	22.0 (7.0, 64.0)	0.10
CSF neutrophil count cells/uL	66	15.5 (6.3, 39.3)	106	13.5 (7.0,36.0)	0.76
CSF lymphocyte count cells/uL	71	33.0 (13.5,71.0)	118	30.0 (11.0,67.8)	0.49
Imaging					
Abnormal Chest X-ray	83	41 (49.4%)	155	87 (56.1%)	0.34
Abnormal CT brain imaging	32	18 (56.3%)	55	32 (58.2%)	1.00
EFA* (95% CI) [log10 CFU/mL of CSF per day]	103	-0.36 (-0.39,-0.33)	187	-0.35 (-0.37,-0.33)	0.49

*EFA = Early Fungal Activity, i.e. the CSF clearance rate of quantitative yeast culture colony counts during the first 14 days of antifungal therapy.

n refers to the number of subjects with non-missing data for that variable. Continuous variables (other than EFA) are summarized as median (interquartile range) and compared using the Wilcoxon rank sum test. Categorical variables are summarized as frequency (percentage) and compared using Fisher’s exact test. EFA was estimated and compared based on mixed effects modeling.

The baseline characteristics between HIV patients infected with ST5 versus non-ST5 organisms were largely similar. However, HIV infected patients with cryptococcal meningitis caused by ST5 organisms were significantly more likely to have lymphadenopathy (11% vs. 4%, *p* = 0.05 Fisher’s exact test) and a higher blood lymphocyte count (median 0.76 versus 0.55 X10^9^ cells/L, *p* = 0.001, Kruskal-Wallis test). CD4 counts tended to be higher in ST5 infected patients, but this was not of statistically significance (median 22 vs. 14.5 cells/uL, *p* = 0.053 Kruskal-Wallis test). Conversely, ST5 infected individuals were less likely to have fungi isolated from blood (61.9% vs. 79.8%, *p* = 0.02 Kruskal-Wallis test) and had lower yeast burdens in CSF at baseline (median 5.4 log10 CFU/ml vs. 5.9 log10 CFU/ml, *p*<0.01 Kruskal-Wallis test). There was no difference in the rate with which yeast was cleared from CSF, defined as early Fungicidal Activity (EFA), by genotype (Table 1).

**Fig 2:**
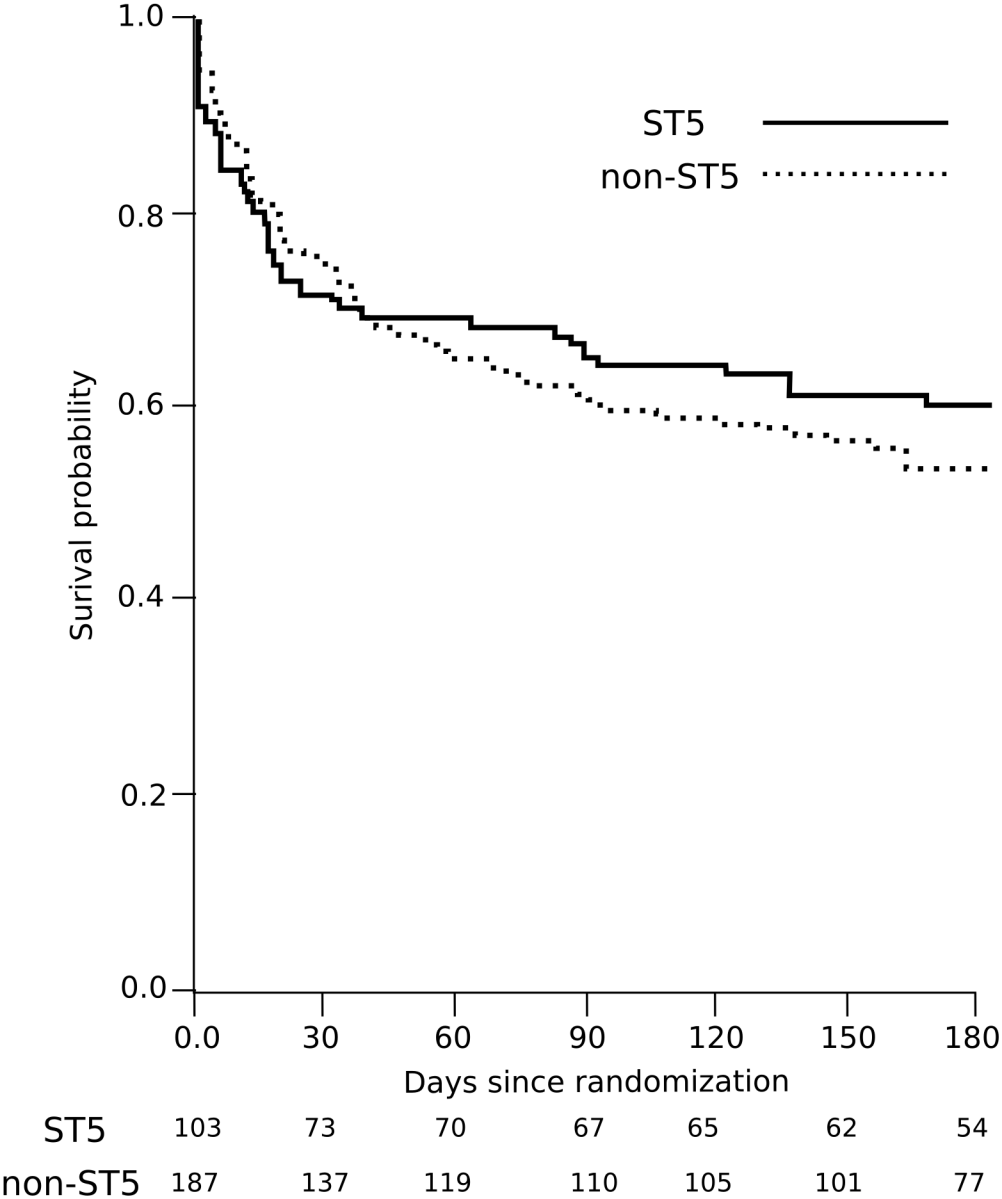
Effect of infection sequence type on survival of Vietnamese HIV infected patients with cryptococcal meningitis. Kaplan-Meier survival curves by infecting sequence type (ST5 (solid line) versus non-ST5 (dashed line)) for 290 HIV infected patients enrolled in a randomized controlled trial of combination antifungal therapy for cryptococcal meningitis over 6 months following randomization. No significant difference in the risk of death was detected by either 10 weeks or 6 months following randomization. Figures below the time axis are the number of patients at risk.

We next investigated the role of ST on disease outcome; the survival curves for cryptococcal infection were similar between the infecting STs (Fig 2). Formal Cox regression analysis (adjusted for treatment assignment) further demonstrated no significant differences in survival up to 70 days or six months post-randomization between patients infected with ST5 or non-ST5 organisms (Hazard Ratio (HR) 0.94 (95%CI 0.62 to 1.44), *p* = 0.72, and HR 0.90 (95%CI 0.62 to 1.31), *p* = 0.57, respectively). These findings were unchanged after further adjustments for baseline fungal burden and Glasgow Coma Score (GCS). However, survivors of ST5 *C*. *neoformans* infections had a borderline significant increased rate of disability at 70 days (72.7% (40/55) in ST5 infections versus 57.1% (52/91) with non-ST5 infections, (OR 2.11 (95%CI 1.01, 4.41), *p* = 0.046).

### Whole genome sequencing of Vietnamese *C. neoformans*

Our results demonstrated that Vietnamese ST5 *C*. *neoformans* organisms are preferentially isolated from HIV non-infected individuals and may induce differing meningitis phenotypes in HIV infected patients. These data suggest that lineage specific genetic loci may be associated with host immune status and/or pathogenicity. To test this hypothesis we selected eight Vietnamese (VN) *C*. *neoformans* var. *grubii* for whole genome sequencing (WGS). The characteristics of the eight strains selected for WGS and the sequencing statistics are shown in Table 2. The selected organisms comprised five ST5 organisms, two ST4 organisms and one ST306 organism and were isolated from both HIV infected and uninfected individuals. The sequencing reads from each of the eight isolates were individually assembled into ~18.3 Mb draft genome assemblies comprising the 14 chromosomes of *C*. *neoformans* var. *grubii*. The assembled sequences had a median scaffold length of 119 kbp with a maximum scaffold length of 464 kbp.

**Table 2:** Characteristics of the eight genome sequenced strains of *Cryptococcus neoformans* var. *grubii*.

Genomic features	BMD700	BMD1338	BMD1646	BK78	BK147	BMD1367	BMD14155	BK80
**Characteristics**								
Underlying Disease	None	None	None	HIV	HIV	Gastric Cancer	SLE	HIV
MLST Sequence type (ST)	5	5	5	5	5	306	4	4
AFLP Cluster	VNIγ	VNIγ	VNIγ	VNIγ	VNIγ	VNIδ	VNIδ	VNIδ
Complication/Outcome	Blind	Died	-	-	-	Died	-	-
**Genome assembly**								
Number of scaffolds	493	506	478	486	503	425	478	466
Total length (Mb)	18.3	18.3	18.3	18.3	18.3	18.3	18.3	18.2
Max length (Kb)	391	391	438	391	391	438	464	438
Min length (Kb)	0.5	0.5	0.5	0.5	0.5	0.5	0.5	0.5
Scaffold N50 (Kb)	111	113	121	126	126	131	115	111
Number of scaffolds larger than N50 size	49	47	44	45	46	41	47	46
Scaffold N90 (Kb)	32	31	35	33	33	38	35	34
Number of scaffolds larger than N90 size	165	163	150	156	157	138	157	157
GC Content (%)	48.2	48.2	48.2	48.2	48.2	48.2	48.2	48.2
Proportion of repetitive sequences (%)	2.11	2.19	2.25	2.20	2.24	2.27	2.30	2.16

The eight draft genome assemblies exhibited approximately 99.4% nucleotide sequence identity with the *C*. *neoformans* var. *grubii* H99 reference sequence. We additionally identified 165 kbp of sequence (0.9% of genome assembly, in fragments >500 bp) specific to either H99 or the VN isolates (Table 3), signifying that the VN organisms were more closely related to each another than to H99. Chromosomal divergence between the eight Vietnamese isolates and the H99 reference strain is illustrated in S1 Fig.

**Table 3:** Alignment of Vietnamese *Cryptococcus neoformans* var. *grubii* genome assemblies against the H99 reference genome.

Genomic features	BMD700	BMD1338	BMD1646	BK78	BK147	BMD1367	BMD1415	BK80
**VN-specific sequences**								
Number of scaffolds	36	34	33	36	35	37	37	35
Total length (Kb)	82.60	86.84	83.94	88.62	88.74	100.52	104.85	97.47
Max length (Kb)	13.72	15.97	15.97	15.97	16.02	14.43	16.01	16.01
Min length (Kb)	0.50	0.50	0.50	0.50	0.50	0.50	0.50	0.50
**H99-specific sequences**								
Number of scaffolds	39	41	40	38	32	75	51	63
Total length (Kb)	64.6	57.16	55.64	55.21	42.24	114.42	75.64	119.97
Max length (Kb)	7.79	3.79	3.77	4.54	3.85	13.62	13.61	11.81
Min length (Kb)	0.58	0.51	0.54	0.52	0.53	0.51	0.51	0.53
Average sequence identity*								
Sequence identity (%)	99.3	99.4	99.4	99.4	99.5	99.3	99.3	99.3

* based on MUMmer show-tiling results

The absence of genetic rearrangements on chromosome 3 and chromosome 11 in VN isolates, in contrast to H99 [24], further suggests that the VN isolates share a more recent common ancestor than strain H99. We next analyzed the mating-type locus in the VN isolates and found that all strains belonged to the most prevalent cryptococcus mating type, MATα.

**Fig 3:**
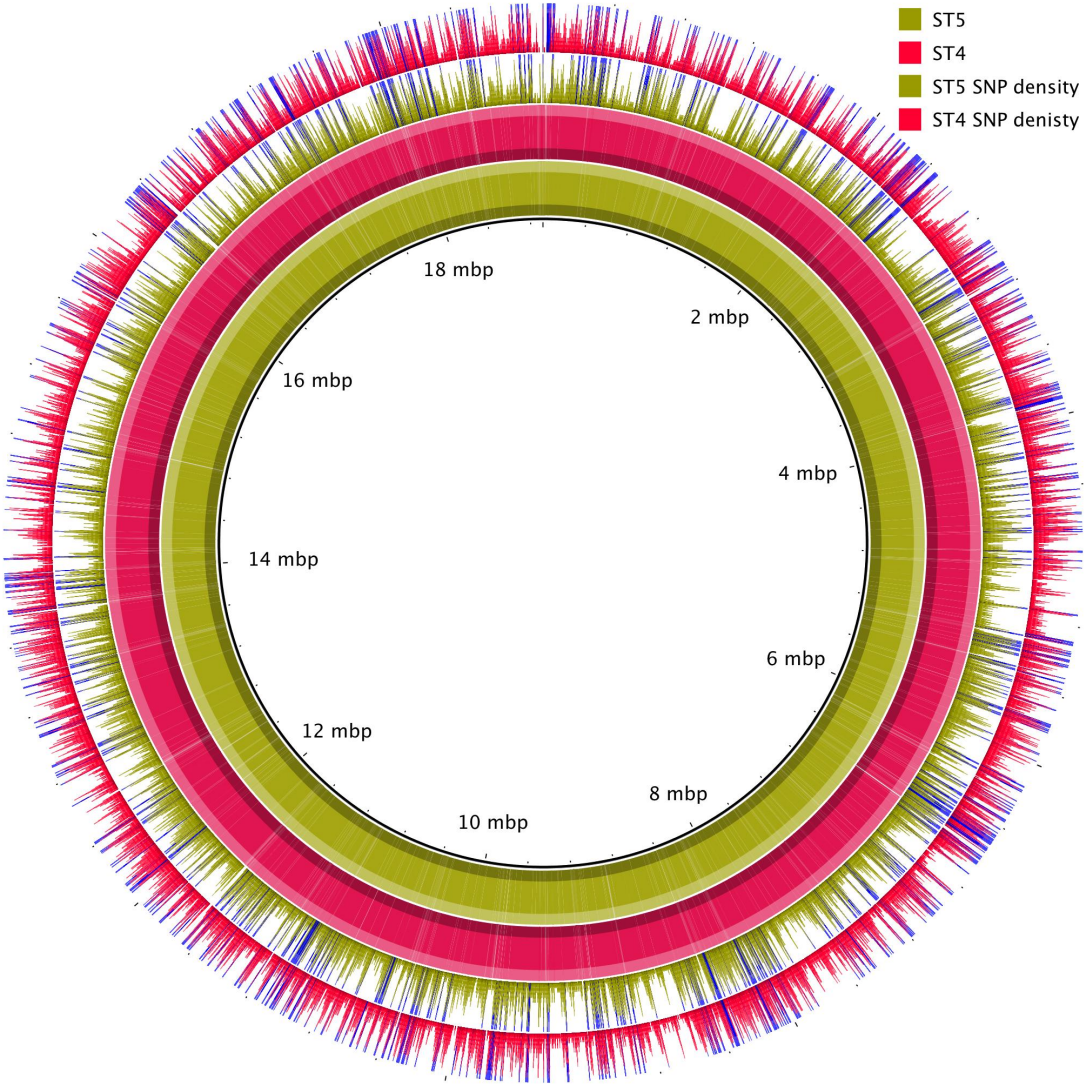
BRIG plot showing the relatedness of an ST5 isolate (BMD700) and an ST4 isolate (BMD1415) to the H99 reference genome. On the inner two rings, the coloured regions represent high pairwise similarity with H99 (>70%) according to BLASTn; lighter regions show areas of difference with H99. The outer two rings plot the number of SNPs per 1000 base pairs. The bar scale is limited to a maximum frequency of 10 SNPs per 1000 bp; any window with greater than this frequency is coloured blue. The figure illustrates that SNP density varies widely across the genome between areas of high and low frequencies; some of these are common to both STs compared with H99, others are ST4 or ST5 specific.

**Fig 4:**
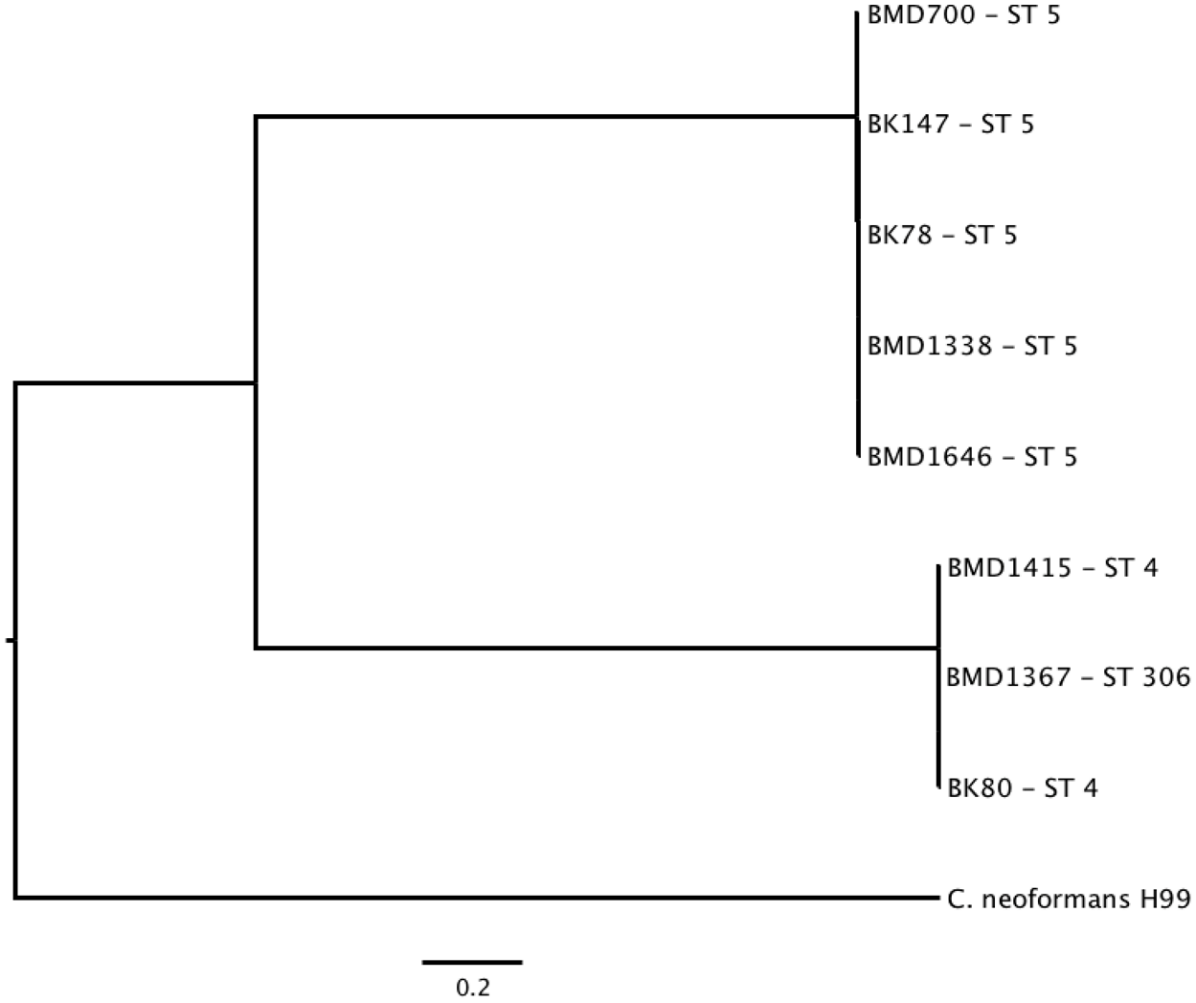
The phylogenetic relationship of eight ST5 and non-ST5 Vietnamese *C*. *neoformans* in comparison with the H99 reference genome. Genome-wide SNP derived maximum likelihood tree of eight Vietnamese strains of *C*. *neoformans* var. *grubii* and the H99 reference strain. Scale bar = genetic distance. All bootstrap values were greater than 0.9.

To infer the phylogenetic relationship between the Vietnamese (VN) isolates, using H99 as a reference, we identified SNPs and indels in the VN strains. We found a mean of 41,290 SNPs and 6,487 indels in each of the VN *C*. *neoformans* var. *grubii* genome assemblies in comparison to H99, and 4,364 indels distinguishing ST5 from non-ST5. SNPs were not evenly distributed throughout the genomes but varied with areas of distinct high frequencies, some shared between lineages and others uniquely associated with ST5 (Fig 3). A neighbor-joining tree confirmed that the ST5 and non-ST5 VN isolates were phylogenetically distinguishable and distantly related to the reference genome (Fig 4).

### Comparative genomics of Vietnamese *C. neoformans*

We next performed comparative genomics to contrast the genome content of the VN *C*. *neoformans* var. *grubii* against H99. The eight VN genomes exhibited approximately 99.7% nucleotide sequence identity, with between 45 kbp (non-ST5) and 67 kbp (ST5) of genotype-specific DNA sequence variation between these genotypes. This was approximately three times less than the extent of the sequence variation between H99 and the VN isolates.

A blastn comparison demonstrated that VN lineage specific DNA was located on multiple chromosomes. Rearrangements and gene loss in *Cryptococcus* are associated with transposable elements and play an integral role in genomic architecture [25, 26]; centromeres are “hotspots” for retrotransposons TCN1 and/or TCN6 [27]. We identified several retrotransposons and repeat elements within the sequences from the VN strains (Table 4). These elements were enriched in chromosomal regions proximal to the telomeres and centromeres. [26]. We found that repeat elements such as the retrotransposons Ty1/Copia and Ty3/Gypsy and the Harbinger interspersed repeat subfamily constituted almost 2% of the VN *C*. *neoformans* genome sequences. The majority of the repeat elements identified in the VN strains belonged to the Ty3/gypsy retrotransposons group (Table 4). Notably, with respect to lineage specific genetic composition, the percentage of repeat elements between ST5/non-ST5 was comparable, with the exception of MuDR DNA transposon sequences, which were found to be more than twice as common in the ST5 isolates (average MuDR repeats; 170 vs. 69 in non-ST5).

**Table 4:** Repeat elements in the Vietnamese *Cryptococcus neoformans* var. *grubii* genome assemblies.

Genomic feature	BMD700	BMD1338	BMD1646	BK78	BK147	BMD1367	BMD1415	BK80
**LTR Retrotransposons**								
Ty1/Copia (bp)	26,115	27,580	26,931	27,172	28,099	25,035	25,382	25,591
Ty3/Gypsy (bp)	191,024	199,833	206,288	201,608	205,351	219,742	223,238	198,448
Others (bp)	37,550	38,648	38,572	38,461	38,170	38,326	38,937	37,663
**DNA elements**								
EnSpm (bp)	373	373	443	443	443	149	97	321
Ginger2/TDD (bp)	54	54	54	54	54	54	54	54
Harbinger (bp)	10,575	10,416	10,448	10,423	10,565	10,481	10,446	103,54
Helitron (bp)	619	619	619	511	619	595	595	595
Mariner/Tc1 (bp)	155	84	155	155	155	146	146	146
MuDR (bp)	158	158	154	158	223	69	69	69
Tcn760 (bp)	101,07	102,09	100,58	101,53	100,53	103,24	103,16	103,93
Others (bp)	365	365	365	365	365	289	289	289
Simple repeats (bp)	86,134	89,912	94,255	90,082	91,273	88,638	89,560	89,625
Low complexity (bp)	6,252	6,332	6,431	6,639	6,687	6,883	7,450	7,074
**Non-LTR Retrotransposon**								
CRE (bp)	16,447	16,784	16,679	17,084	17,576	13,785	14,405	13,728
Tad1 (bp)	130	68	68	68	68	0	0	65

To test our hypothesis of lineage-specific genetic variation accounting for phenotypic distinction in HIV/non-HIV infected patients we performed comparative genomics between the ST5 and the non-ST5 sequences. The ST5 and non-ST5 specific DNA sequences were *de novo* assembled; forming 45 contiguous sequences (25 in ST5, 20 in non-ST5); these are described in Table 5. These genotype specific sequences ranged from 0.5 to 20 kbp and contained between one and six predicted coding sequences. The 45 genotype specific regions were subjected to blastx/p to identify homologous and orthologous DNA and protein sequences. The majority of these genotype-specific sequences encoded cryptococcal proteins with multiple homologues across the *C*. *neoformans* var. *grubii* genome (S2 Table). As many of these homologues were likely to share functional redundancy we focused on non-redundant lineage specific coding sequences for further investigation.

**Table 5:** Characteristics of the Vietnamese *Cryptococcus neoformans* var. *grubii* sequences unique to either ST5 or non-ST5 isolates. Lineage specific sequence was highly conserved within each lineage.

**Genome features**	**Sequences unique to ST5**					**Sequences unique to non-ST5**		
	**BMD700**	**BMD1338**	**BMD1646**	**BK78**	**BK147**	**BMD1367**	**BMD1415**	**BK80**
Number of scaffolds	25	28	25	26	25	21	23	20
Total length (Kb)	63.13	66.44	65.32	66.85	65.29	50.08	53.24	44.56
Max length (Kb)	19.99	19.99	19.99	19.99	19.99	6.09	7.18	5.50
Min length (Kb)	0.51	0.51	0.50	0.51	0.51	0.50	0.52	0.50
	**Compared with non-ST5**					**Compared with ST5**		
Average sequence identity*	**BMD700**	**BMD1338**	**BMD1646**	**BK78**	**BK147**	**BMD1367**	**BMD1415**	**BK80**
Sequence identity (%)	99.9	99.9	99.3	99.8	99.3	99.9	99.9	99.9

* based on MUMmer show-tiling results

The specific sequences found in the ST5 strains encoded 19 predicted proteins with a previously annotated function and eight hypothetical proteins. Notably, with the exception of a fungal-specific phenolic acid decarboxylase (PAD) previously identified in *Meyerozyma guilliermondii*, all ST5 genotype-specific sequences could be found in *C*. *neoformans* H99 [28]. ST5-specific genes encoding proteins potentially associated with virulence included a DEAD-box ATP-dependent RNA helicase 26 (CNAG_07651), oxoprolinases (implicated in host colonization), a taurine catabolism dioxygenase (stress resistance), a zinc finger protein, membrane transport proteins and drug transporters [29].

We additionally found that the ST5 specific phenolic acid decarboxylase specific gene (PAD) was located on a DNA scaffold that could be aligned to the telomeric region of chromosome 11. This region was adjacent to a non-LTR retrotransposon Cnl1 (*C*. *neoformans* LINE-1) encoding region; we conclude this was a likely insertion into the ST5 genome. The DNA sequence of the PAD gene was found to be sufficiently divergent (blastx E-value = 3e-16) from that of other fungi PAD genes (*Meyerozyma guilliermondii* PAD, sequence homology for 30 of the 168 aa) and overall GC content of the scaffold did not differ substantially from that of the overall genome sequence. Taken together these data suggest that this insertion event was not recent [28]. We confirmed the presence of PAD in a publically available ST5 strain from South Africa (SRA sequence accession ERR1810411).

In the context of the Vietnamese cohort in which they were isolated non-ST5 specific sequences potentially represent ‘anti-virulence’ encoding regions, since they are not present in ST5 organisms [30]. The non-ST5 specific DNA sequences (n = 20) included regions encoding three hypothetical proteins (CNAG_07666, CNAG_00127 and CNAG_07313), a sugar transporter (CNAG_06527), a heat shock protein and *TAR1* (temperature associated repressor gene; CNAG_04934) [31, 32]. CNAG_07666 was found to contain a domain related to the CAP (cysteine-rich secretory proteins, antigen 5, and pathogenesis-related 1 proteins) family and exhibited homology to the pathogenesis-related protein, Pr-1. However, atypically for proteins within the CAP family, CNAG_07666 was found to have only a single cysteine residue, and at 16 kDa was predicted to be only half the size of other previously described crypotococcal Pr-1 type proteins [33]. CNAG_00127 was predicted to encode a hypothetical protein with no known homologues and no previously described protein domains or motifs. The region encoding the CNAG_00127 protein was partially deleted in all of the sequenced ST5 isolates, and the proportion of gene deleted across the different ST5 isolates was variable [31]. TAR1 inhibits the expression of melanin at 37°C, and has previously been shown to be a potential virulence factor for *Cryptococcus* [32]. We confirmed the presence of TAR1 encoding sequences in the genome assemblies of non-ST5 isolates by blastn and blastx (sequences exhibited 100% DNA sequence identity with strain H99). Temperature dependent gene expression may represent an advantageous host adaptation and therefore such genes are potentially associated with virulence. Two further regions encoding enzymes with temperature dependent expression (allantoate permease CNAG_06875 (ST5-specific) and aldo-keto reductase CNAG_01257 (non-ST5 specific)) were also found to be genotype specific [34]. We additionally found that a region encoding a sodium/bile acid cotransporter (CNAG_01461) protein was partially truncated in the non-ST5 strains (381 aa instead of 515 aa) in comparison to the H99 reference. The majority of proteins encoded by genotype-specific DNA were predicted to have enzymatic function, but given their high sequence homology to other homologues and orthologues in the genome it was difficult to infer their significance and function in genetic variation.

In addition to genotype-specific sequence, we identified a number of genotype-specific SNPs (in relation to the H99 reference genome) likely to significantly impact protein function because they resulted in premature truncation of translation (Table 6). In the ST5 strains these SNPs were found to be located in three previously identified virulence associated proteins; a cytosine-purine permease (CNAG_04982) expressed during macrophage infection, a temperature dependent hypothetical protein (CNAG_06731) and an Ire1protein kinase (CNAG_03670) [35–37]. SNPs resulting in premature gene truncation occurred in a further 16 ST5-specific genes included those encoding a calcineurin-like phosphoesterase and nine hypothetical proteins. Similarly, a number of such SNPs were identified in the non-VNIg strains. Twenty-five genes were affected, four which have previously been identified as virulence determinants, including two expressed during macrophage infection (CNAG_01464 and CNAG_01445) and two with temperature dependent ((37°C) expression (CNAG_01257 and CNAG_03754) [35, 36]. Thirteen of the 25 affected genes were conserved hypothetical proteins; a further three were predicted proteins.

**Table 6:** Genes containing genotype-specific SNPs and indels specific to the Vietnamese *Cryptococcus neoformans* var. *grubii* ST5 or non-ST5 organism.

Gene ID	Sequence type	High impact mutations (n)	Protein name	Pfam Accession number	Pfam description
CNAG_07704	ST5	32	Conserved hypothetical protein		
CNAG_02339	ST5	11	Conserved hypothetical protein		
CNAG_05161	ST5	8	Conserved hypothetical protein		
CNAG_05185	ST5	5	Conserved hypothetical protein		
CNAG_06888	ST5	4	Cytoplasmic protein	PF00400.23	WD domain, G-beta repeat. (protein-protein interaction)
CNAG_04921	ST5	3	Conserved hypothetical protein		
CNAG_05987	ST5	3	Conserved hypothetical protein		
CNAG_00005	ST5	2	TPR domain-containing protein	PF07719.8	Tetratricopeptide repeat. (protein-protein interaction)
CNAG_01240	ST5	2	Conserved hypothetical protein		
CNAG_01964	ST5	2	Oligopeptide transporter	PF03169.6	OPT oligopeptide transporter protein
CNAG_02027	ST5	2	Conserved hypothetical protein		
CNAG_02968	ST5	2	phospholipase C-2		
CNAG_06251	ST5	2	ser/thr protein phosphatase family protein	PF00149.19	Calcineurin-like phosphoesterase
CNAG_06338	ST5	2	ABC transporter PMR5	PF00005.18	ABC transporter
CNAG_06503	ST5	2	Uridine permease	PF02133.6	Permease for cytosine/purines, uracil, thiamine, allantoin
CNAG_06810	ST5	2	Conserved hypothetical protein		
CNAG_04982*	ST5	1	Cytosine-purine permease		
CNAG_06731*	ST5	1	Conserved hypothetical protein		
CNAG_03670*	ST5	1	Other/IRE protein kinase	PF00069.16	Protein kinase domain
CNAG_04773	Non-ST5	23	Conserved hypothetical protein		
CNAG_06867	Non-ST5	19	Conserved hypothetical protein		
CNAG_03189	Non-ST5	10	DIL and Ankyrin domain-containing protein	PF00023.21	Ankyrin repeat
CNAG_06934	Non-ST5	10	Hexose transporter protein	PF07690.7	Major Facilitator Superfamily
CNAG_07682	Non-ST5	8	Conserved hypothetical protein	PF04886.3	PT repeat
CNAG_05328	Non-ST5	7	Conserved hypothetical protein	PF00172.9	Fungal Zn(2)-Cys(6) binuclear cluster domain
CNAG_00174	Non-ST5	5	Conserved hypothetical protein		
CNAG_00642	Non-ST5	5	Conserved hypothetical protein		
CNAG_01891	Non-ST5	4	RAD57 protein (DNA repair)		
CNAG_02475	Non-ST5	3	Flavin-containing monooxygenase		
CNAG_07727	Non-ST5	3	Predicted protein		
CNAG_01240	Non-ST5	2	Conserved hypothetical protein		
CNAG_01866	Non-ST5	2	Conserved hypothetical protein		
CNAG_02478	Non-ST5	2	Glycerol dehydrogenase	PF00248.12	Aldo/keto reductase family
CNAG_05064	Non-ST5	2	Conserved hypothetical protein		
CNAG_05185	Non-ST5	2	Conserved hypothetical protein		
CNAG_06307	Non-ST5	2	Conserved hypothetical protein		
CNAG_07312	Non-ST5	2	Conserved hypothetical protein		
CNAG_07815	Non-ST5	2	Conserved hypothetical protein		
CNAG_07832	Non-ST5	2	Predicted protein		
CNAG_07928	Non-ST5	2	Predicted protein		
CNAG_01464*	Non-ST5	1	flavohemoglobin	PF00175.12	Oxidoreductase NAD-binding domain
CNAG_01445*	Non-ST5	1	APG9	PF04109.7	Autophagy protein Apg9
CNAG_01257*	Non-ST5	1	aldo-keto reductase	PF00248.12	Aldo/keto reductase family

* previously described virulence associated genes

## Discussion

Although cryptococcal meningitis due to infection with *C*. *neoformans* var. *grubii* is predominantly a disease of immunocompromised patients, disease in the apparently immunocompetent is increasingly recognized in Asia [5, 38, 39]. Our analyses demonstrate that our previously defined VNIγ cluster, responsible for the vast majority of disease in HIV uninfected patients in Vietnam, consists of a single MLST type (ST5), and that the divergence of this genotype from other Vietnamese strains is not recent [6]. In contrast, clinical isolates from HIV infected patients in Vietnam appear to be more diverse, with at least 14 different STs found to cause disease in this group. However, while we have demonstrated clear segregation of strains according to host immune phenotype, ST5 strains are also the single most frequent cause of disease in HIV infected patients, accounting for >30% of cases. The dominance of ST5 strains in HIV infected patients could be explained by increased abundance of this ST in the environment, leading to more exposure and opportunity for infection. Alternatively, this ST may have enhanced pathogenic potential compared with other STs, predicting it has a greater inherent capacity to cause infections in humans. In the latter case, its prevalence in the HIV infected population would be expected to be more common than its prevalence in the environment. The dominance of ST5 strains in immunocompetent patients, the relative low incidence of this disease, together with the low HIV prevalence in Vietnam (1%) is consistent with a hypothesis of increased pathogenicity and low environmental prevalence. Systematic and sensitive randomized environmental sampling is needed to test this hypothesis.

Despite the differential segregation of strains by host immune status, the clinical data from HIV patients does not suggest that ST5 strains are more virulent in this patient group. Although patients with ST5 infections tended to have lower levels of consciousness, which have previously been associated with worse outcomes, we did not find significant differences in rates of death, and only marginal differences in disability in survivors, by infecting genotype [8]. This finding is similar to a study from South Africa, which also did not identify statistically significant differences in outcome in HIV infected patients according to infecting sequence type [40]. Surprisingly, the burden of fungus in the CSF, which has previously been identified as an important prognostic factor, was significantly lower in patients infected with ST5 strains [41]. We found no difference in duration of symptoms between the ST5 induced infections and the non-ST5 infections. Therefore, the lower fungal burdens associated with ST5 infections are unlikely to be a consequence of earlier presentation of a more severe illness. An alternative interpretation is that ST5 strains are more ‘potent’ on a cell-by-cell basis, leading to similar clinical outcomes despite lower yeast burdens. Consistent with the previously identified immune segregation of strains, there was a trend towards higher CD4 counts in HIV patients infected with ST5. Consequently, while it seems that, in HIV patients at least, the infecting genotype does not have a major impact on disease course, ST5 strains may have an advantage in either the colonization or invasion of hosts. Pathogenicity factors that confer these abilities are not well defined although we did identify differences in genes between strains that have previously been associated with macrophage infection. The genetic differences between ST5 and the other strains in genes encoding hypothetical and predicted proteins are intriguing prospects for further study in experimental models with respect to these qualities.

Cryptococcal disease is not transmitted person to person, and humans are a dead-end host. Therefore, the drivers for genetic divergence must be related to *C*. *neoformans*’ (as yet unidentified) ecological niche in Vietnam. The ability to cause disease is thought to be a by-product of such adaptations—so called ‘bystander pathogenicity’ [6]. Recognized ecological niches for *Cryptococcus* species in other geographic locations include bird guano, soil, rotting wood and various tree species [2]. The presence of a novel phenolic acid decarboxylase (PAD) gene associated exclusively with the ST5 strains may provide evidence for the adaptation of these strains to a particular niche in Vietnam. Phenolic acids are important lignin-related constituents of plant cell walls, and therefore prevalent in the environment of *C*. *neoformans*. Cell wall-bound phenolic acids interfere with cell wall degrading enzymes and mycelia growth of fungi; the acquisition of this PAD may have been positively selected to combat plant defenses [42]. Of note, depending on the fungal growth medium, phenolic acid can also be incorporated into melanin, which is known to be an important *Cryptococcus* virulence factor [43]. The acquisition of the PAD gene presumably represents a horizontal gene transfer event, possibly from a closely related member of the *Cryptococcus* species or an alternative fungal species inhabiting the same niche. Notably, we found Cnl1 retrotransposon elements adjacent to this gene, likely indicating their role in the insertion of this gene [44]. However, the Cnl1 element was incomplete, suggesting this gene is defunct and the gene is now fused into the genome following transposition. This scenario resembles a previous report of interspecies gene transfer between fungi [45]. More studies are necessary to determine whether PAD is functional in ST5 isolates and whether it plays a significant role in virulence. However, in our isolates, the vast majority of genotype-specific DNA encoded previously identified known cryptococcal associated proteins, suggesting that the loss of cryptococcal genes is of greater evolutionary significance for disease than the acquisition of genes supporting novel functions [46]. Evidently, the loss and acquisition of genetic material we observed here is limited to those that do not affect survival outside the host. Gene loss can be tolerated because of functional redundancy between similar proteins. Further, where there may be only a single copy of a gene, there may be pleiotropic effects such that genes have both an essential housekeeping function as well as playing a key role in virulence [47].

We found differences in the presence and absence of numerous temperature dependent genes between the ST5 and non-ST5 isolates. Such genes enable some limited adaptation towards the environment in the susceptible human and are important virulence candidates. Of particular interest was the deletion of the TAR1 gene in the ST5 isolates. TAR1 was initially reported to inhibit laccase expression in a temperature-associated manner, resulting in reduced production of melanin at 37°C [32]. Therefore, this gene could be considered to have an antivirulence function in humans. TAR1 has been reported to have a small but significant attenuating effect in a cryptococcal mouse infection model [48]. Such disabled antivirulence mechanisms are not novel—they have been reported as a mechanism of increased virulence in several bacterial pathogens, where they may have been acquired for adaptation to the environment [30]. However, the deletion of TAR1 is unlikely to be sufficient to explain the extent of the clinical differences between ST5 and other non-ST5 isolates. Moreover, the regulatory effects of TAR1 seem to vary by strain, indicating that melanin production is controlled through multiple pathways [48].

Further potential cause of differences in pathogenicity in *Cryptococcus* are genomic rearrangements with consequent gene disruptions or altered expression of adjacent genes due to transposon insertions, excision events, gene deletions, duplications, inversions and translocation events due to ectopic recombination [26]. We were unable to explicitly test gene duplication using our data given the limitations of short read Illumina sequence data. These limitations also mean large-scale rearrangements would not have been apparent in the draft genome assemblies. There is a higher probability of DNA rearrangement occurring in fungi exposed to environmental stresses, the acquisition of which presumably offers an evolutionary advantage in a specific ill-defined niche [49]. In our isolates, the location of novel genotype-specific DNA showed a telomeric bias, possibly due to alternative selective constraints at the telomeres or because of neighboring gene co-expression [50]. DNA rearrangements may also influence recombination between the various genotypes, which can contribute to the “speciation” of new genotypes [51, 52].

A potential weakness of our study is that we sequenced and typed only single isolates from our patients. Thus, we may have missed mixed lineage infections. However given the association between ST5 strains and HIV uninfected patients is so statistically robust it is unlikely that the distribution we see is an artefact [6].

In conclusion, we have performed comparative genomics and a clinical comparison of *Cryptococcus neoformans* var. *grubii* isolates and shown that ST5 and non-ST5 strains have a comparable genetic content, despite significantly different ability to induce disease in non-HIV infected individuals. Our analysis identified a number of gene candidates for further study to disaggregate the pathways associated with the pathogenesis of *Cryptococcus neoformans*. While we found little difference in the outcome of disease in HIV infected patients according to infecting sequence type, this is in contrast with the asymmetrical distribution of sequence types seen in clinical practice according to host immune phenotype. Therefore we postulate that the genetic differences identified between strains in this study in some way result in different abilities in effecting either host colonization, invasion, or latency. Currently we lack robust models of disease in immunocompetent patients for these important phases of infection, but *ex vivo* gene expression studies, particularly from patients with different immune phenotypes, are likely to be more revealing and offer the prospect of identifying novel drug targets.

## Supporting information

S1 Fig Chromosomal synteny between 8 Vietnamese clinical isolates of *Cryptococcus neoformans* and the H99 reference. Figure generated using Mauve, http://darlinglab.org/mauve/mauve.html.
(TIF)

S1 Table Clinical characteristics of 136 clinical isolates of *C*. *neoformans*. (DOCX)

S2 Table Proteins encoded by genotype specific sequence (ST5 versus non-ST5). (DOCX)

S1 Methods TAR1 PCR conditions. (DOCX)
