# Supplementary figures and images for: Comparative genomics of Cryptococcus neoformans var. grubii associated with meningitis in HIV infected and uninfected patients in Vietnam
Source: PLoS Negl Trop Dis. 2017 Jun 14;11(6):e0005628. doi: 10.1371/journal.pntd.0005628 (PMC5484541; doi:10.1371/journal.pntd.0005628)

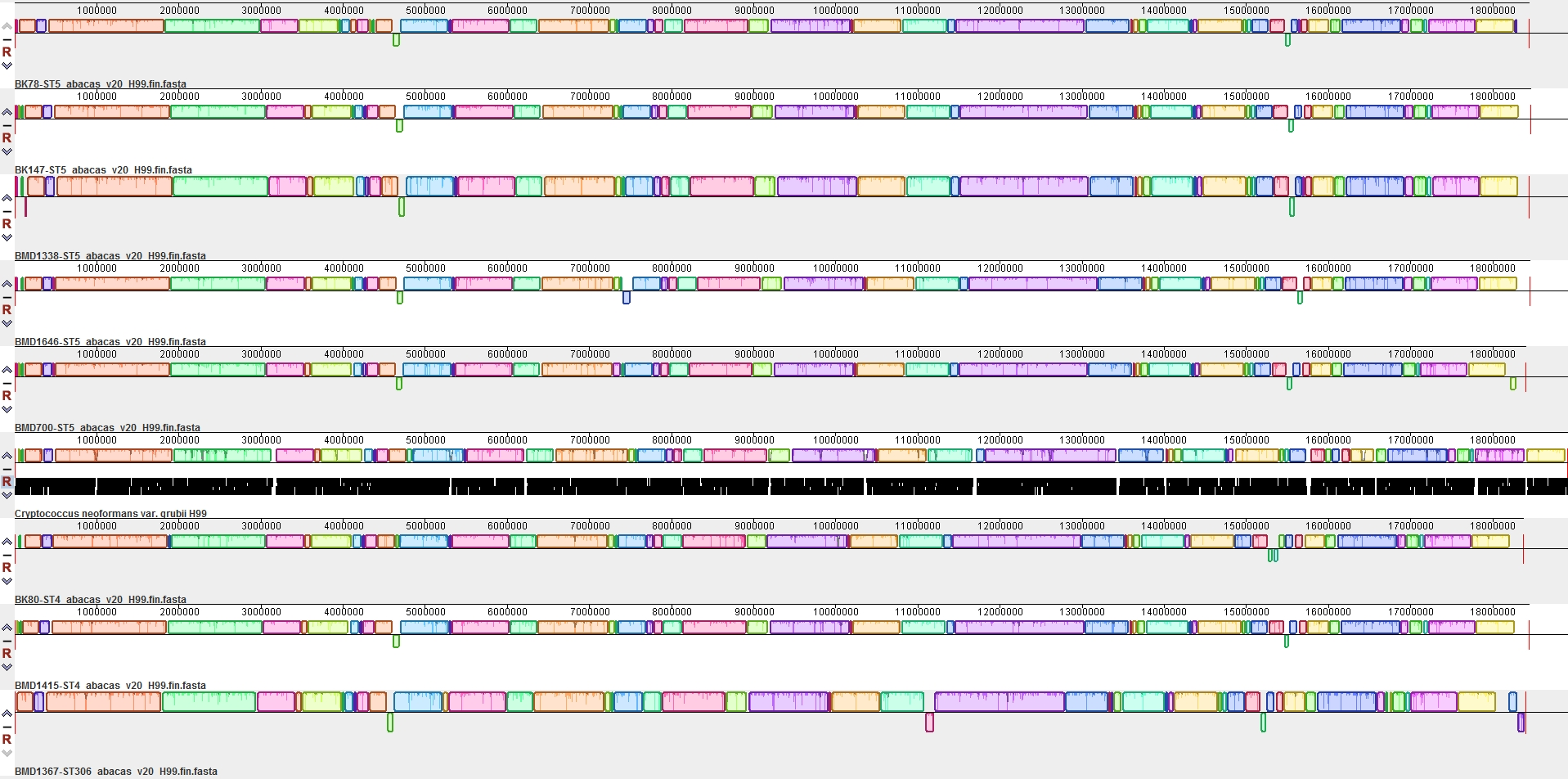

Supplement: S1 Fig — Figure generated using Mauve, http://darlinglab.org/mauve/mauve.html. (TIF) [file pntd.0005628.s001.tif]
