# Supplementary material for: Comparative genomics of Cryptococcus neoformans var. grubii associated with meningitis in HIV infected and uninfected patients in Vietnam
Source: PLoS Negl Trop Dis. 2017 Jun 14;11(6):e0005628. doi: 10.1371/journal.pntd.0005628 (PMC5484541; doi:10.1371/journal.pntd.0005628)
Supplement: S1 Table — (DOCX) [file pntd.0005628.s002.docx]

**Supporting Information Table S1:**

**Clinical Characteristics of 136 clinical isolates of *C. neoformans* var. *grubii***

| **Isolate number** | **HIV status** | **Year isolated** | **MLST AT** | **MLST ST** | **AFLP Type** | **Sequenced Y/N** |
| --- | --- | --- | --- | --- | --- | --- |
| VNBK001 | Infected | 2004 | 1421151 | 4 | VNIdelta | N |
| VNBK002 | Infected | 2004 | 1421151 | 4 | VNIdelta | N |
| VNBK004 | Infected | 2004 | 1523111 | 5 | VNIgamma | N |
| VNBK011 | Infected | 2004 | 13411110 | 32 | VNIdelta | N |
| VNBK012 | Infected | 2004 | 134231110 | 93 | VNIdelta | N |
| VNBK014 | Infected | 2004 | 1421151 | 4 | VNIdelta | N |
| VNBK015 | Infected | 2004 | 1523111 | 5 | VNIgamma | N |
| VNBK017 | Infected | 2004 | 13411110 | 32 | VNIdelta | N |
| VNBK018 | Infected | 2004 | 1321151 | 6 | VNIdelta | N |
| VNBK020 | Infected | 2004 | 1523111 | 5 | VNIgamma | N |
| VNBK023 | Infected | 2004 | 1421151 | 4 | VNIdelta | N |
| VNBK024 | Infected | 2004 | 134231110 | 93 | VNIdelta | N |
| VNBK025 | Infected | 2004 | 1523111 | 5 | VNIgamma | N |
| VNBK026 | Infected | 2004 | 1523111 | 5 | VNIgamma | N |
| VNBK027 | Infected | 2004 | 13411110 | 32 | VNIdelta | N |
| VNBK028 | Infected | 2004 | 1523111 | 5 | VNIgamma | N |
| VNBK030 | Infected | 2004 | 1421151 | 4 | VNIdelta | N |
| VNBK033 | Infected | 2004 | 134231110 | 93 | VNIdelta | N |
| VNBK034 | Infected | 2004 | 1523111 | 5 | VNIgamma | N |
| VNBK035 | Infected | 2004 | 1421151 | 4 | VNIdelta | N |
| VNBK038 | Infected | 2005 | 1523111 | 5 | VNIgamma | N |
| VNBK041 | Infected | 2005 | 1523111 | 5 | VNIgamma | N |
| VNBK042 | Infected | 2005 | 1523111 | 5 | VNIgamma | N |
| VNBK044 | Infected | 2005 | 1523111 | 5 | VNIgamma | N |
| VNBK045 | Infected | 2005 | 1523111 | 5 | VNIgamma | N |
| VNBK046 | Infected | 2005 | 13411110 | 32 | VNIdelta | N |
| VNBK048 | Infected | 2005 | 1421151 | 4 | VNIdelta | N |
| VNBK049 | Infected | 2005 | 1523111 | 5 | VNIgamma | N |
| VNBK050 | Infected | 2005 | 1523111 | 5 | VNIgamma | N |
| VNBK052 | Infected | 2005 | 1321151 | 6 | VNIdelta | N |
| VNBK054 | Infected | 2005 | 1523111 | 5 | VNIgamma | N |
| VNBK055 | Infected | 2005 | 1328231110 | 340 | VNIdelta | N |
| VNBK056 | Infected | 2005 | 1421151 | 4 | VNIdelta | N |
| VNBK057 | Infected | 2005 | 1421151 | 4 | VNIdelta | N |
| VNBK058 | Infected | 2005 | 1523111 | 5 | VNIgamma | N |
| VNBK059 | Infected | 2005 | 1421151 | 4 | VNIdelta | N |
| VNBK062 | Infected | 2005 | 1523111 | 5 | VNIgamma | N |
| VNBK063 | Infected | 2005 | 1523111 | 5 | VNIgamma | N |
| VNBK064 | Infected | 2005 | 1521151 | 188 | VNIdelta | N |
| VNBK068 | Infected | 2005 | 13411110 | 32 | VNIdelta | N |
| VNBK069 | Infected | 2005 | 1421151 | 4 | VNIdelta | N |
| VNBK071 | Infected | 2005 | 1321151 | 6 | VNIdelta | N |
| VNBK073 | Infected | 2005 | 1421151 | 4 | VNIdelta | N |
| VNBK074 | Infected | 2005 | 1421151 | 4 | VNIdelta | N |
| VNBK075 | Infected | 2005 | 1321151 | 6 | VNIdelta | N |
| VNBK076 | Infected | 2005 | 1523111 | 5 | VNIgamma | N |
| VNBK78 | Infected | 2005 | 1523111 | 5 | VNIgamma | Y |
| VNBK80 | Infected | 2005 | 1421151 | 4 | VNIdelta | Y |
| VNBK085 | Infected | 2005 | 134231110 | 93 | VNIdelta | N |
| VNBK087 | Infected | 2005 | 1421151 | 4 | VNIdelta | N |
| VNBK088 | Infected | 2005 | 1421151 | 4 | VNIdelta | N |
| VNBK089 | Infected | 2005 | 1421151 | 4 | VNIdelta | N |
| VNBK090 | Infected | 2005 | 1421151 | 4 | VNIdelta | N |
| VNBK094 | Infected | 2005 | 1523111 | 5 | VNIgamma | N |
| VNBK096 | Infected | 2005 | 1321151 | 6 | VNIdelta | N |
| VNBK109 | Infected | 2006 | 1423151 | 137 | VNIdelta | N |
| VNBK111 | Infected | 2006 | 1421151 | 4 | VNIdelta | N |
| VNBK115 | Infected | 2006 | 1523111 | 5 | VNIgamma | N |
| VNBK116 | Infected | 2006 | 1523111 | 5 | VNIgamma | N |
| VNBK117 | Infected | 2006 | 1523111 | 5 | VNIgamma | N |
| VNBK119 | Infected | 2006 | 1523111 | 5 | VNIgamma | N |
| VNBK120 | Infected | 2006 | 1421151 | 4 | VNIdelta | N |
| VNBK124 | Infected | 2006 | 1523111 | 5 | VNIgamma | N |
| VNBK129 | Infected | 2006 | 154231110 | 195 | VNIdelta | N |
| VNBK139 | Infected | 2006 | 1523111 | 5 | VNIgamma | N |
| VNBK147 | Infected | 2006 | 1523111 | 5 | VNIgamma | Y |
| VNBK150 | Infected | 2006 | 13431110 | 338 | VNIdelta | N |
| VNBK151 | Infected | 2006 | 1421151 | 4 | VNIdelta | N |
| VNBK153 | Infected | 2006 | 15251110 | 339 | VNIdelta | N |
| VNBK154 | Infected | 2006 | 13411111 | 39 | VNIdelta | N |
| VNBK156 | Infected | 2006 | 1421151 | 4 | VNIdelta | N |
| VNBK157 | Infected | 2006 | 134231110 | 93 | VNIdelta | N |
| VNBK160 | Infected | 2006 | 1523111 | 5 | VNIgamma | N |
| VNBK163 | Infected | 2006 | 1421151 | 4 | VNIdelta | N |
| VNBK167 | Infected | 2006 | 13411111 | 39 | VNIdelta | N |
| VNBK169 | Infected | 2007 | 1523111 | 5 | VNIgamma | N |
| VNBK171 | Infected | 2007 | 1523111 | 5 | VNIgamma | N |
| VNBK172 | Infected | 2007 | 1521151 | 188 | VNIdelta | N |
| VNBK175 | Infected | 2007 | 1523111 | 5 | VNIgamma | N |
| VNBK179 | Infected | 2007 | 134231110 | 93 | VNIdelta | N |
| VNBK182 | Infected | 2007 | 1421151 | 4 | VNIdelta | N |
| VNBK185 | Infected | 2007 | 1423111 | 337 | VNIgamma | N |
| VNBK188 | Infected | 2007 | 1321151 | 6 | VNIdelta | N |
| VNBK189 | Infected | 2007 | 1321151 | 6 | VNIdelta | N |
| VNBK190 | Infected | 2007 | 1523111 | 5 | VNIgamma | N |
| VNBK192 | Infected | 2007 | 1421151 | 4 | VNIdelta | N |
| VNBK193 | Infected | 2007 | 1421151 | 4 | VNIdelta | N |
| VNBK205 | Infected | 2007 | 1321151 | 6 | VNIdelta | N |
| VNBK209 | Infected | 2008 | 13411111 | 39 | VNIdelta | N |
| VNBK213 | Infected | 2008 | 134231110 | 93 | VNIdelta | N |
| VNBK218 | Infected | 2008 | 1321151 | 6 | VNIdelta | N |
| VNBK219 | Infected | 2008 | 1321151 | 6 | VNIdelta | N |
| VNBK224 | Infected | 2008 | 1421151 | 4 | VNIdelta | N |
| VNBK225 | Infected | 2008 | 1421151 | 4 | VNIdelta | N |
| VNBK228 | Infected | 2008 | 134231110 | 93 | VNIgamma | N |
| VNBK234 | Infected | 2009 | 1321151 | 6 | VNIdelta | N |
| VNBK241 | Infected | 2009 | 1523111 | 5 | VNIgamma | N |
| BMD101 | Uninfected | 1997 | 1523111 | 5 | VNIgamma | N |
| BMD367 | Uninfected | 2000 | 1523111 | 5 | VNIgamma | N |
| BMD368 | Uninfected | 2000 | 1523111 | 5 | VNIgamma | N |
| BMD394 | Uninfected | 2000 | 1421151 | 4 | VNIgamma | N |
| BMD494 | Uninfected | 2001 | 1523111 | 5 | VNIgamma | N |
| BMD534 | Uninfected | 2001 | 1523111 | 5 | VNIgamma | N |
| BMD673 | Uninfected | 2002 | 1523111 | 5 | VNIgamma | N |
| BMD700 | Uninfected | 2002 | 1523111 | 5 | VNIgamma | Y |
| BMD732 | Uninfected | 2003 | 1523111 | 5 | VNIgamma | N |
| BMD745 | Uninfected | 2003 | 1321151 | 6 | VNIgamma | N |
| BMD761 | Uninfected | 2003 | 1523111 | 5 | VNIgamma | N |
| BMD852 | Uninfected | 2004 | 1523111 | 5 | VNIgamma | N |
| BMD854 | Uninfected | 2005 | 1523111 | 5 | VNIgamma | N |
| BMD865 | Uninfected | 2005 | 1523111 | 5 | VNIgamma | N |
| BMD894 | Uninfected | 2006 | 1523111 | 5 | VNIgamma | N |
| BMD899 | Uninfected | 2006 | 1523111 | 5 | VNIgamma | N |
| BMD903 | Uninfected | 2006 | 1523111 | 5 | VNIgamma | N |
| BMD910 | Uninfected | 2006 | 1523111 | 5 | VNIgamma | N |
| BMD915 | Uninfected | 2006 | 13411110 | 32 | VNIdelta | N |
| BMD942 | Uninfected | 2006 | 13411110 | 32 | VNIdelta | N |
| BMD973 | Uninfected | 2006 | 1523111 | 5 | VNIgamma | N |
| BMD1198 | Uninfected | 2007 | 1523111 | 5 | VNIgamma | N |
| BMD1228 | Uninfected | 2007 | 1523111 | 5 | VNIgamma | N |
| BMD1232 | Uninfected | 2007 | 1523111 | 5 | VNIgamma | N |
| BMD1291 | Uninfected | 2007 | 1523111 | 5 | VNIgamma | N |
| BMD1338 | Uninfected | 2007 | 1523111 | 5 | VNIgamma | Y |
| BMD1353 | Uninfected | 2007 | 1523111 | 5 | VNIgamma | N |
| BMD1367 | Uninfected | 2007 | 30321151 | 306 | VNIdelta | Y |
| BMD1392 | Uninfected | 2007 | 1421151 | 4 | VNIdelta | N |
| BMD1415 | Uninfected | 2007 | 1421151 | 4 | VNIdelta | Y |
| BMD1452 | Uninfected | 2008 | 1523111 | 5 | VNIgamma | N |
| BMD1465 | Uninfected | 2008 | 1523111 | 5 | VNIgamma | N |
| BMD1534 | Uninfected | 2008 | 1523111 | 5 | VNIgamma | N |
| BMD1592 | Uninfected | 2008 | 1523111 | 5 | VNIgamma | N |
| BMD1646 | Uninfected | 2008 | 1523111 | 5 | VNIgamma | Y |
| BMD1713 | Uninfected | 2008 | 1523111 | 5 | VNIgamma | N |
| BMD1716 | Uninfected | 2008 | 1523111 | 5 | VNIgamma | N |
| BMD1828 | Uninfected | 2009 | 1523111 | 5 | VNIgamma | N |
| BMD1879 | Infected | 2009 | 1421151 | 4 | VNIdelta | N |
