# Supplementary material for: Comparative genomics of Cryptococcus neoformans var. grubii associated with meningitis in HIV infected and uninfected patients in Vietnam
Source: PLoS Negl Trop Dis. 2017 Jun 14;11(6):e0005628. doi: 10.1371/journal.pntd.0005628 (PMC5484541; doi:10.1371/journal.pntd.0005628)
Supplement: S2 Table — (DOCX) [file pntd.0005628.s003.docx]

**Supporting Information Table S2:**

**Proteins encoded by genotype specific sequence (ST5 versus non-ST5)**

| **MLST** | **Scaffold No.** | **Scaffold size (Kb)** | **specific DNA size (Kb)** | **Genes encoded** |
| --- | --- | --- | --- | --- |
| **non-ST5** | s_6_C178566 | 4.94 | 4.8 | flavin-containing monooxygenase (C. gattii) |
|  | s_6_C178710 | 6.09 | 6.09 | MFS alpha-glucoside transporter (Cordyceps militaris) |
|  |  |  |  | dehydrogenase (C. neoformans) |
|  | s_6_scaffold2493 | 11.06 | 1.09 | - |
|  | s_6_scaffold3308 | 7.77 | 2.74 | hypothetical protein (C. gattii) |
|  |  |  |  | hypothetical protein (C. gattii) |
|  | s_6_C178922 | 7.67 | 2.93 | L-idonate 5-dehydrogenase (C. gattii) |
|  |  |  |  | myo-inositol transporter 1 (C. gattii) |
|  | s_6_scaffold1123 | 9.71 | 5.5 | polyprotein (*Puccinia graminis*) |
|  | s_6_scaffold140 | 7.83 | 1.25 | reverse transcriptase (C. neoformans) |
|  |  |  | 2.76 | retrotransposon nucleocapsid protein (C. neoformans) |
|  | s_6_scaffold148 | 1.53 | 1.33 | hypothetical protein (C. neoformans) |
|  | s_6_scaffold1597 | 159.69 | 0.66 | DnaJ/Hsp40 protein (C. neoformans) |
|  | s_6_scaffold23 | 19.27 | 2.6 | putative pathogenesis-related protein (Oryza sativa) |
|  | s_6_scaffold4799 | 4.79 | 4.27 | glycerol dehydrogenase (C. gattii) |
|  |  |  |  | dehydrogenase (C. neoformans) |
|  | s_6_scaffold5372 | 2.44 | 1.7 | MFS monocarboxylate transporter (Talaromyces stipitatus) |
|  | s_6_scaffold73 | 205.22 | 1.69 | temperature associater repressor (C. grubii) |
|  | s_6_C177378 | 1.1 | 1.1 | reverse transcriptase (C. neoformans) |
|  | s_6_C176718 | 0.74 | 0.74 | retrotransposon nucleocapsid protein (C. neoformans) |
| **ST5** | **Scaffold No.** | **Scaffold size (Kb)** | **VNIγ-specific DNA size (Kb)** | **Protein encoded** |
|  | s_1_C359806 | 1.56 | 1.49 | hypothetical protein (C. neoformans) |
|  | s_1_scaffold2868 | 2.05 | 1.84 | integrase-reverse transcriptase (C. neoformans) |
|  | s_1_scaffold3267 | 4.99 | 1.59 | hypothetical protein (C. neoformans) |
|  | s_1_scaffold59 | 4.22 | 2.36 | hypothetical protein (C. gattii) |
|  |  |  |  | hypothetical protein (C. gattii) |
|  | s_1_scaffold3389 | 2.54 | 1.55 | reverse transcriptase (C. neoformans) |
|  | s_1_C359594 | 1.18 | 1.18 | retrotransposon nucleocapsid protein (C. neoformans) |
|  | s_1_C359660 | 1.3 | 1.3 | *hypothetical protein (C. neoformans) |
|  | s_1_scaffold4319 | 4.56 | 0.5 | *hypothetical protein (C. neoformans) |
|  |  |  | 2.64 | *hypothetical protein (C. neoformans) |
|  | s_1_scaffold3024 | 27.48 | 1.37 | hypothetical protein (C. gattii) |
|  | s_1_scaffold103 | 36.08 | 11.8 | 5-oxoprolinase (C. neoformans) |
|  |  |  |  | D-galactonate transporter (C. neoformans) |
|  |  |  |  | probable taurine catabolism dioxygenase (C. neoformans) |
|  | s_1_scaffold212 | 48.03 | 0.53 | reverse transcriptase (C. neoformans) |
|  | s_1_scaffold263 | 20.13 | 19.99 | amidohydrolase (C. gattii) |
|  |  |  |  | zinc-finger protein (C. gattii) |
|  |  |  |  | D-galactonate transporter (C. neoformans) |
|  |  |  |  | drug transporter (C. gattii) |
|  |  |  |  | aminotransferase (C. gattii) |
|  |  |  |  | hypothetical protein (C. gattii) |
|  | s_1_scaffold4325 | 27.75 | 1.38 | fungal specific phenolic acid decarboxylase (Meyerozyma guilliermondii) |
|  |  |  | 0.7 | membrane transport protein (C. gattii) |
|  | s_1_scaffold36 | 150.65 | 0.54 | MULE transposase domain-containing protein (C. neoformans) |
|  | s_1_scaffold3759 | 44.66 | 3.77 | alcohol dehydrogenase (C. neoformans) |
|  | s_1_scaffold38 | 47.97 | 2.17 | DEAD-box RNA helicase (C. neoformans) |
|  | s_1_scaffold732 | 2.57 | 0.93 | helicase (C. neoformans) |
