# Supplementary material for: Comparative genomics of Cryptococcus neoformans var. grubii associated with meningitis in HIV infected and uninfected patients in Vietnam
Source: PLoS Negl Trop Dis. 2017 Jun 14;11(6):e0005628. doi: 10.1371/journal.pntd.0005628 (PMC5484541; doi:10.1371/journal.pntd.0005628)
Supplement: S1 Methods — (DOCX) [file pntd.0005628.s004.docx]

**Supporting Information Methods S1**

**TAR1 PCR:**

PCR of the *TAR1* gene was conducted in a final volume of 50 μL. Each reaction contained 50 ng of DNA, 1X PCR buffer (10 mM Tris-HCl, pH 8.3, 50 mM KCl, 1.5 mM MgCl2; Applied Biosystems, Foster City, CA), 0.4 mM each of dATP, dTTP, dGTP and dCTP (Roche Diagnostics GmbH), 3 mM magnesium acetate, 1.5 U AmpliTaq DNA polymerase (Applied Biosystems), and 50 ng of each primer TAR1 5’-CACGAATTGGGACAGGAAGT-3’ and 5’-GAAGAGAAGGAGGCGGAACT-3’. PCR was performed for 35 cycles in a Perkin-Elmer model 480 thermal cycler under the following conditions: 94°C for 2 minutes denaturation then 45 s denaturation at 94°C, annealing at 60°C for 60 seconds; extension for 2-minutes at 72°C, then final extension at 72°C for 10 minutes.
